# Cardiac autonomic impairment and chronotropic incompetence in fibromyalgia

**DOI:** 10.1186/ar3519

**Published:** 2011-11-18

**Authors:** Roberta Potenza da Cunha Ribeiro, Hamilton Roschel, Guilherme Gianini Artioli, Thalita Dassouki, Luiz Augusto Perandini, Ana Luisa Calich, Ana Lúcia de Sá Pinto, Fernanda Rodrigues Lima, Eloísa Bonfá, Bruno Gualano

**Affiliations:** 1 Division of Rheumatology, School of Medicine, University of Sao Paulo, Brazil. Av. Dr. Arnaldo, 455, Cerqueira César, Brazil; 2 School of Physical Education and Sport, University of Sao Paulo, Brazil. Av. Prof. Mello Moraes, 65, Butantã, Brazil

**Keywords:** abnormal heart-rate response, cardiovascular risk, autonomic dysfunction

## Abstract

**Introduction:**

We aimed to gather knowledge on the cardiac autonomic modulation in patients with fibromyalgia (FM) in response to exercise and to investigate whether this population suffers from chronotropic incompetence (CI).

**Methods:**

Fourteen women with FM (age: 46 ± 3 years; body mass index (BMI): 26.6 ± 1.4 kg/m^2^) and 14 gender-, BMI- (25.4 ± 1.3 kg/m^2^), and age-matched (age: 41 ± 4 years) healthy individuals (CTRL) took part in this cross-sectional study. A treadmill cardiorespiratory test was performed and heart-rate (HR) response during exercise was evaluated by the chronotropic reserve. HR recovery (deltaHRR) was defined as the difference between HR at peak exercise and at both first (deltaHRR1) and second (deltaHRR2) minutes after the exercise test.

**Results:**

FM patients presented lower maximal oxygen consumption (VO_2 _max) when compared with healthy subjects (22 ± 1 *versus *CTRL: 32 ± 2 mL/kg/minute, respectively; *P *< 0.001). Additionally, FM patients presented lower chronotropic reserve (72.5 ± 5 *versus *CTRL: 106.1 ± 6, *P *< 0.001), deltaHRR1 (24.5 ± 3 *versus *CTRL: 32.6 ± 2, *P *= 0.059) and deltaHRR2 (34.3 ± 4 *versus *CTRL: 50.8 ± 3, *P *= 0.002) than their healthy peers. The prevalence of CI was 57.1% among patients with FM.

**Conclusions:**

Patients with FM who undertook a graded exercise test may present CI and delayed HR recovery, both being indicative of cardiac autonomic impairment and higher risk of cardiovascular events and mortality.

## Introduction

Fibromyalgia (FM) is a chronic syndrome characterized by widespread pain and discomfort. In general, FM is accompanied by other symptoms such as fatigue, sleep disorders, reduced muscular strength and endurance, parasthesis, irritable bowel and joint stiffness [1]. Over the last two decades, the understanding regarding FM physiopathology has substantially advanced, with a growing body of evidence suggesting that autonomic nervous system dysfunction (also called dysautonomia) plays a role in this disease [2-4].

In this regard, Furlan *et al. *[5] showed that women with FM have exacerbated sympathetic activity at rest. Furthermore, FM women undertaking a tilt test displayed an inability to increase sympathetic discharge and decrease vagal activity. Accordingly, dysautonomia has also been noticed in response to standing and cold exposure [6], both well-known autonomic function stressors.

Importantly, autonomic nervous system dysfunction has been associated with higher cardiovascular risk in several populations [7,8]. In this regard, heart rate (HR) response to a graded exercise test has been largely used for assessing risks and prognoses in patients with overt or subclinical cardiovascular diseases [9,10]. For example, an attenuated HR response to exercise (that is, chronotropic incompetence (CI))-which results from the combination of parasympathetic withdrawn and sympathetic activation-has been considered a strong and independent predictor of mortality and coronary heart disease, even when controlled for age, physical fitness, standard cardiovascular risk factors and ST-segment changes with exercise [9,10]. Kingsley *et al. *[11] evaluated for the first time autonomic modulation in women with FM after an acute bout of resistance exercise. The authors contended that patients had greater parasympathetic and lower sympathetic modulation than healthy controls. However, the authors did not evaluate autonomic control during exercise, precluding a more definitive conclusion about their findings [11]. In this respect, Van Denderen *et al. *1992 observed attenuated (nor)epinephrine concentrations during a graded exercise until exhaustion in FM patients *versus *healthy controls, suggesting a disturbance in autonomic control.

Thus, we aimed to gather knowledge on the cardiac autonomic modulation in FM patients in response to exercise and to investigate whether this population suffers from CI.

## Materials and methods

### Study design and patients

This was a cross-sectional case-control study. The sample consisted of 14 women with primary FM (age: 46 ± 3 years; BMI: 26.6 ± 1.4 kg/m^2^) and 14 gender-, BMI- (25.4 ± 1.3 kg/m^2^), and age-matched (41 ± 4 years) healthy individuals (CTRL). All patients fulfilled the revised American College of Rheumatology preliminary diagnostic criteria for FM [12].

Exclusion criteria were: *i) *cardiovascular involvement (for example, arrhythmias, arterial hypertension, heart failure, conduction disturbances, myocarditis and pericarditis); *ii) *tobacco usage; *iii*) use of chronotropic and antihypertensive drugs; *iii) *other chronic diseases (for example, systemic lupus erythematosus, rheumatoid arthritis, diabetes mellitus, dyslipidemia and chronic kidney disease). At entry, patients were taking analgesics (50.0%), anti-depressive drugs (35.7%), muscle relaxants (35.7%), nonsteroidal anti-inflammatory drugs (21.4%), and anticonvulsants (7.4%) in stable doses for at least three months. Control subjects were not taking any medication. Both patients and controls were not engaged in regular physical activity programs for the past six months prior to the study.

The study was approved by the Local Ethical Committee and all of the subjects signed the written informed consent.

### Cardiorespiratory exercise testing

A modified Balke treadmill (Centurion, model 200, Micromed, Brasília, Brazil) maximal exercise test [13] was used to assess the cardiac autonomic modulation in patients with FM in response to exercise. Oxygen consumption (VO_2_) and carbon dioxide output were obtained through breath-by-breath sampling and expressed as a 30-s average using an indirect calorimetry system (Cortex-model Metalyzer III B, Leipzig, Germany). HR was continuously recorded at rest, during exercise and at recovery, using a 12-lead electrocardiogram (Ergo PC Elite, InC. Micromed, Brasília, Brazil). The recovery period was set at two minutes using the initial workload (1.9 mph). Maximal oxygen consumption (VO_2 _max), ventilatory anaerobic threshold (VAT) and respiratory compensation point (RCP) were determined according to previously described criteria [14].

### HR response during exercise and recovery

HR response during exercise was evaluated by the chronotropic reserve, as follows: (chronotropic reserve = (peak HR-resting HR/220-age-resting HR) × 100) [15]. CI was determined when subjects failed to achieve < 80% of the chronotropic reserve, despite reaching a peak exercise respiratory exchange ratio (RER) > 1.05 (which suggests adequate effort) [15]. HR recovery (deltaHRR) was defined as the difference between HR at peak exercise and at both first (deltaHRR1) and second (deltaHRR2) minutes after exercise.

### Statistical analysis

Data are presented as mean ± S.E.M. Unpaired Student *t*-tests were used to assess differences between groups for all of the dependent variables. Absolute change was used to calculate the difference between the HR at peak exercise and at the first and second minutes after the exercise test. Significance level was previously set at *P *< 0.05.

## Results

All subjects achieved RER > 1.05 and volitional exhaustion. Significant differences were found in VO_2 _max (FM: 22 ± 1 vs. CTRL: 32 ± 2 mL/kg/min, *P *< 0.01), peak HR (FM: 148 ± 5 *vs*. CTRL: 177 ± 3 bpm, *P *< 0.001), HR at RCP (FM: 133 ± 4 *vs*. CTRL: 177 ± 5 bpm, *P *< 0.001).

**Figure 1:**
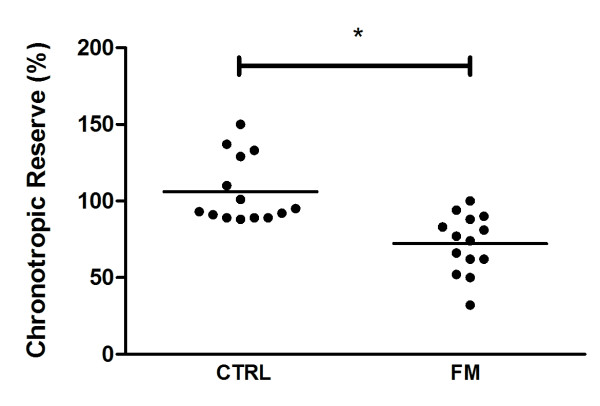
**Chronotropic reserve during exercise in patients with fibromyalgia (FM) and healthy controls (CTRL)**. Data are expressed as mean and individual data. * *P *< 0.001 *vs*. controls.

**Figure 2:**
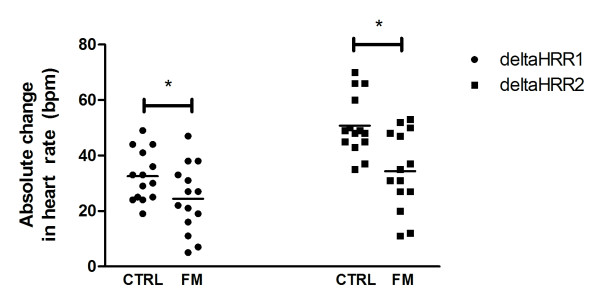
**Changes in heart-rate recovery at the first (deltaHRR1) and second (deltaHRR2) minute of recovery**. Horizontal bars represent the means. * indicates *P *= 0.059 and *P *= 0.002 when compared with CTRL at deltaHRR1 and delta HRR2, respectively.

Chronotropic reserve was significantly lower for the patients with FM when compared with CTRL (72.5 ± 5 *vs*. 106.1 ± 6, respectively; *P *< 0.001) (Figure 1). Eight of the 14 patients with FM (57.1%) presented CI whereas none of the healthy subjects presented it. deltaHRR1 (FM: 24.5 ± 3 *vs*. CTRL: 32.6 ± 2 bpm; *P *= 0.059) and deltaHRR2 (FM: 34.3 ± 4 *vs*. CTRL: 50.8 ± 3; *P *= 0.002) were also lower in FM versus CTRL (Figure 2, panels A and B, respectively).

## Discussion

This study indicates that patients with FM present abnormal HR response during and after exercise. In addition, we have identified that 57.1% of the patients presented CI, which is a powerful predictor of all-cause and cardiac death [7,8].

CI has been recognized as a predictor of outcome in several cohorts that have included tens of thousands of patients [10,16]. For instance, Sandvik *et al. *[10] observed that the HR increase during exercise was negatively associated with survival and that a reduction in the HR increase was a strong predictor of mortality from cardiovascular disease in a cohort of apparently healthy men. We observed a similar abnormal chronotropic response, suggesting that FM patients may also have a higher risk of mortality when compared with their healthy matched controls.

The mechanisms underlying CI during exercise still remain elusive. However, the most likely explanation for abnormal HR response involves abnormalities in autonomic nervous system modulation [17]. Several studies using power spectrum analysis of HR variability with different stressors (for example, tilt table test and 24 h electrocardiogram registration) have showed increased sympathetic and decreased parasympathetic tones in FM patients [2-4]. Interestingly, this autonomic dysfunction appears to be characterized by sustained sympathetic hyperactivity at rest, and hyporeactivity to stress [1]. In this context, the blunted HR response during exercise seen in FM patients may be related to the desensitization of cardiac β1 receptors through a heightened sympathetic activity similar to heart failure [17]. Longitudinal studies are necessary to test the predictive power of CI as a non-invasive marker of risk in patients with FM.

In addition to the sympathetic hypoactivity observed during maximal exercise, cardiac autonomic impairment was noted following exercise, as suggested by a delayed deltaHRR recovery, which is initially affected by parasympathetic reactivation followed by sympathetic withdrawal. Altogether, these findings suggest that dysautonomia may play a role in FM. However, additional studies using more direct measurements of autonomic nervous system (for example, microneurography) may provide further insights on dysautonomia in FM.

Importantly, autonomic dysfunction has been associated with a variety of symptoms in FM, including sleep disorders, chronic pain, allodynia, anxiety, pseudo-Raynaud's phenomenon, sicca syndrome and intestinal irritability [18]. Thus, strategies aimed to attenuate dysautonomia may be considered therapeutically promising in FM. In this regard, Figueroa *et al. *[2] showed that a 16-week resistance training program led to improvements in resting vagal modulation and pain perception in women with FM. Additionally, the same group demonstrated that aerobic exercise training may improve post-exercise autonomic modulation in obese and type 2 diabetic patients [19]. Long-term studies should explore the therapeutic role of different types of exercise training (for example, low- or high-intensity, resistance, endurance or combined exercise programs) in modulating autonomic nervous system (for example, delayed HR recovery) in FM. Whether or not exercise-induced autonomic modulation may ameliorate CI in FM patients is also unclear.

Even though we have excluded all subjects taking drugs which affect chronotropic response to exercise (for example, beta-blockers), it is important to note that our patients were under a variety of medications (for example, antidepressant drugs), which may somehow affect the autonomic system [20]. Nonetheless, we extensively reviewed the literature and found no evidence of an influence of such drugs on chronotropic reserve or HR recovery. Accordingly, *post-hoc *sub-group analyses revealed that patients not taking antidepressants also had delayed HR recovery when compared with paired controls (deltaHRR1 = -13 bpm; *P *= 0.02 and deltaHRR1 = -20 bpm; *P *= 0.002). Moreover, five of nine patients (55.5%) not using antidepressant drugs presented CI. This prevalence was similar to that observed in subjects altogether (57.1%), suggesting that these drugs played a minor (if any) role in the current findings. Further studies assessing patients free of medication may be needed to fully clarify this issue.

Considering that both RER and HR during exercise are dependent on the volitional effort of the person exercising, one may speculate that a submaximal effort may explain the low HR responses in 57% of the FM patients tested. However, all of the patients reported volitional exhaustion at the end of the test. This, along with the fact that a RER greater than 1.05 was achieved in all tests, allows us to believe that CI may actually have accounted for low HR responses seen in this study.

## Conclusions

Patients with FM presented an abnormal HR response to exercise, suggesting cardiac autonomic impairment and higher risk of cardiac events and mortality.

## Key messages

• Patients with fibromyalgia present cardiac autonomic impairment in response to exercise.

• Chronotropic incompetence and delayed heart-rate recovery may predispose fibromyalgic patients to a higher mortality risk.

## Abbreviations

BMI: body mass index; CI: chronotropic incompetence; CTRL: healthy subjects; deltaHRR: heart rate recovery; deltaHRR1: heart rate recovery at the first minute; deltaHRR2: heart rate recovery at the second minute; FM: fibromyalgia; HR: heart rate; RCP: respiratory compensation point; RER: respiratory exchange ratio; VAT: ventilatory anaerobic threshold; VO_2_: oxygen consumption; VO_2 _max: maximal oxygen consumption.

## Competing interests

The authors declare that they have no competing interests.

## Authors' contributions

HR, RPCR and BG were responsible for concept and design, statistical expertise, data analysis and interpretation, and helped write the manuscript. FRL was responsible for data analysis and interpretation and helped write the manuscript. GGA and ALSP were significant manuscript reviewers/revisers and were responsible for data analysis and interpretation. TD and ALC were significant manuscript reviewers/revisers and were responsible for data acquisition. LAP was a significant manuscript reviewer/reviser and was responsible for data acquisition, analysis and interpretation. EB was a significant manuscript reviewer/reviser and was responsible for concept and design. All authors have read and approved the manuscript for publication.

## References

[B1] Di Franco M Iannuccelli C Valesini G Neuroendocrine immunology of fibromyalgia Ann N Y Acad Sci 2010 1193 84 90 10.1111/j.1749-6632.2009.05344.x 20398012

[B2] Figueroa A Kingsley JD McMillan V Panton LB Resistance exercise training improves heart rate variability in women with fibromyalgia Clin Physiol Funct Imaging 2008 28 49 54 18005081 10.1111/j.1475-097X.2007.00776.x

[B3] Martinez-Lavin M Hermosillo AG Mendoza C Ortiz R Cajigas JC Pineda C Nava A Vallejo M Orthostatic sympathetic derangement in subjects with fibromyalgia J Rheumatol 1997 24 714 718 9101507

[B4] Raj SR Brouillard D Simpson CS Hopman WM Abdollah H Dysautonomia among patients with fibromyalgia: a noninvasive assessment J Rheumatol 2000 27 2660 2665 11093450

[B5] Furlan R Colombo S Perego F Atzeni F Diana A Barbic F Porta A Pace F Malliani A Sarzi-Puttini P Abnormalities of cardiovascular neural control and reduced orthostatic tolerance in patients with primary fibromyalgia J Rheumatol 2005 32 1787 1793 16142879

[B6] Qiao ZG Vaeroy H Morkrid L Electrodermal and microcirculatory activity in patients with fibromyalgia during baseline, acoustic stimulation and cold pressor tests J Rheumatol 1991 18 1383 1389 1757941

[B7] Tsuji H Larson MG Venditti FJ Jr Manders ES Evans JC Feldman CL Levy D Impact of reduced heart rate variability on risk for cardiac events. The Framingham Heart Study Circulation 1996 94 2850 2855 8941112 10.1161/01.cir.94.11.2850

[B8] Tsuji H Venditti FJ Jr Manders ES Evans JC Larson MG Feldman CL Levy D Reduced heart rate variability and mortality risk in an elderly cohort. The Framingham Heart Study Circulation 1994 90 878 883 8044959 10.1161/01.cir.90.2.878

[B9] Nishime EO Cole CR Blackstone EH Pashkow FJ Lauer MS Heart rate recovery and treadmill exercise score as predictors of mortality in patients referred for exercise ECG JAMA 2000 284 1392 1398 10.1001/jama.284.11.1392 10989401

[B10] Sandvik L Erikssen J Ellestad M Erikssen G Thaulow E Mundal R Rodahl K Heart rate increase and maximal heart rate during exercise as predictors of cardiovascular mortality: a 16-year follow-up study of 1960 healthy men Coron Artery Dis 1995 6 667 679 10.1097/00019501-199508000-00012 8574463

[B11] Kingsley JD Panton LB McMillan V Figueroa A Cardiovascular autonomic modulation after acute resistance exercise in women with fibromyalgia Arch Phys Med Rehabil 2009 90 1628 1634 10.1016/j.apmr.2009.02.023 19735793

[B12] Wolfe F Clauw DJ Fitzcharles MA Goldenberg DL Katz RS Mease P Russell AS Russell IJ Winfield JB Yunus MB The American College of Rheumatology preliminary diagnostic criteria for fibromyalgia and measurement of symptom severity Arthritis Care Res (Hoboken) 2010 62 600 610 10.1002/acr.20140 20461783

[B13] Braga A Nunes N Negrão CE, Baretto ACP Avaliação cardiopulmonar Cardiologia do Exercício: do Atleta ao Cardiopata 2005 1 São Paulo, Brazil: Manole 128 147

[B14] Howley ET Bassett DR Jr Welch HG Criteria for maximal oxygen uptake: review and commentary Med Sci Sports Exerc 1995 27 1292 1301 8531628

[B15] Brubaker PH Kitzman DW Chronotropic incompetence: causes, consequences, and management Circulation 2011 123 1010 1020 10.1161/CIRCULATIONAHA.110.940577 21382903 PMC3065291

[B16] Lauer MS Francis GS Okin PM Pashkow FJ Snader CE Marwick TH Impaired chronotropic response to exercise stress testing as a predictor of mortality JAMA 1999 281 524 529 10.1001/jama.281.6.524 10022108

[B17] Lauer MS Chronotropic incompetence: ready for prime time J Am Coll Cardiol 2004 44 431 432 10.1016/j.jacc.2004.05.001 15261943

[B18] Martinez-Lavin M Hermosillo AG Autonomic nervous system dysfunction may explain the multisystem features of fibromyalgia Semin Arthritis Rheum 2000 29 197 199 10.1016/S0049-0172(00)80008-6 10707988

[B19] Figueroa A Baynard T Fernhall B Carhart R Kanaley JA Endurance training improves post-exercise cardiac autonomic modulation in obese women with and without type 2 diabetes Eur J Appl Physiol 2007 100 437 444 10.1007/s00421-007-0446-3 17406886

[B20] Licht CM de Geus EJ van Dyck R Penninx BW Longitudinal evidence for unfavorable effects of antidepressants on heart rate variability Biol Psychiatry 2010 68 861 868 10.1016/j.biopsych.2010.06.032 20843507

